# Antibacterial activities of the extracts, fractions and isolated compounds from *Canarium patentinervium* Miq. against bacterial clinical isolates

**DOI:** 10.1186/s12906-020-2837-5

**Published:** 2020-02-14

**Authors:** R. Mogana, A. Adhikari, M. N. Tzar, R. Ramliza, C. Wiart

**Affiliations:** 1 Department of Pharmaceutical Biology, Faculty of Pharmaceutical Sciences, UCSI University, No. 1 Jln Menara Gading, UCSI Heights, 56000 Cheras, Kuala Lumpur, Malaysia; 2 Central Department of Chemistry, Tribhuvan University, Kritipur, Kathmandu, Nepal; 3 Department of Medical Microbiology and Immunology, Hospital National University of Malaysia, Cheras, Kuala Lumpur, Malaysia; 4 School of Pharmacy, Faculty of Science, Center for Natural and Medicinal Products Research, University of Nottingham (Malaysia Campus), Jalan Broga, 43500 Semenyih, Selangor Malaysia

**Keywords:** *Canarium patentinervium* Miq., Bacterial clinical isolates, MIC, MBC, Hyperin, Cynaroside

## Abstract

**Background:**

*Canarium patentinervium* leaves are used by the local indigenous people of Malaysia for wound healing. The current study is undertaken to screen the comprehensive antibacterial activity of the leaves and barks extracts, fractions and isolated compounds from this plant. Bioassay guided fractionation was also undertaken to deeply evaluate the antibacterial activity of the water fraction of the leaves extract. This is to provide preliminary scientific evidence to the ethnopharmacology usage of this plant by investigating antibacterial properties of the plant and its isolated constituents.

**Methods:**

Bio-assay guided fractionation and subsequent isolation of compounds using open column chromatography. The antibacterial activity against gram positive and gram negative ATCC strain and resistant clinical strains were evaluated using microtiter broth dilution method to determine minimum inhibitory concentration (MIC), minimum bactericidal concentration (MBC) and time-kill assay. The chemical structure of the isolated compounds from the water fraction of the ethanol extract of leaves was elucidated using Nuclear Magnetic Resonance (NMR).

**Results:**

The ethanol extract of the leaves and barks showed antimicrobial activity against all four ATCC and eight clinical isolates. The ethanol extract of the leaves and the corresponding water fraction had good activity against MRSA *S. aureus.* (MIC: 250 μg/ml) and had bactericidal effect on eight of the clinical strains (MSSA,MRSA, oxacillin-resistant CONS, oxacillin-sensitive CONS, *Enterococcus faecalis*, *Klebsiela* species*, Kleb pneumoniae* ESBL and *Candida parapsilosis*). Further phytochemical investigation of the water fraction of the crude ethanol extract of leaves afforded compound 7 (hyperin) and compound 8 (cynaroside) that had bactericidal activity against tested bacterial species (MIC 50 μg/ml and 100 μg/ml). The two compounds were isolated from this genus for the first time.

**Conclusions:**

These results may provide a rational support for the traditional use of *Canarium patentinervium* Miq. in infections and wound healing, since the antimicrobial compounds isolated were also present in the leaves extract.

## Background

Since their introduction, antimicrobials (antibiotics) have played an essential role in decreasing morbidity due to infectious diseases. However infectious diseases remain the leading cause of death worldwide and bacteria have become more resistant to conventional antibiotic in recent years. The widespread use of these compounds is thought to further encourage the emergence of antimicrobial resistance. Lord Jim O’Neill and his team published a high profile review report in 2014 that estimated that antimicrobial resistance (AMR) could cause 10 million deaths a year by 2050 [1]. This phenomenon of increased drug resistance, combined with the multiplicity of side effects by existing agents and the emergence of diseases for which no treatment yet exists, makes the search for the new antimicrobial agents a highly relevant and important subject for research. The number of resistant pathogenic bacteria grows at an alarming rate worldwide and the search for novel antimicrobial agents from medicinal plants to combat such pathogens has become crucial for avoiding the emergence of untreatable bacterial infections [2, 3]. For centuries, plants have been used in the traditional treatment of microbial infections. This assembly of knowledge by indigenous peoples about plants and their products continue to play an essential role in health care of a great proportion of the population [4].

Burseraceae Kunth is one of the nine flowering plant families belonging to the order Sapindales Juss. ex Bercht. & J. Pearl that comprise the monophyletic group (5700 species), whose first known fossils appear in Europe 65 million years ago (Ma). The Burseraceae Kunth. consists of approximately 18 genera and 700 species of which the genus *Canarium* L. embraces 75 species of trees which are mainly found in tropical Asia and the Pacific, and a few species in tropical Africa [5], about 9 species were found in the Philippines [6]. Only an alarming 12% of the 75 species have been studied for their pharmacological activities. *C. patentinervium* Miq. is a rare plant from the family of Burseraceae and genus *Canarium* found in Asia Pacific region previously recorded for its usage in wound healing by the indigenous people of Malaysia [6].

**Fig. 1:**
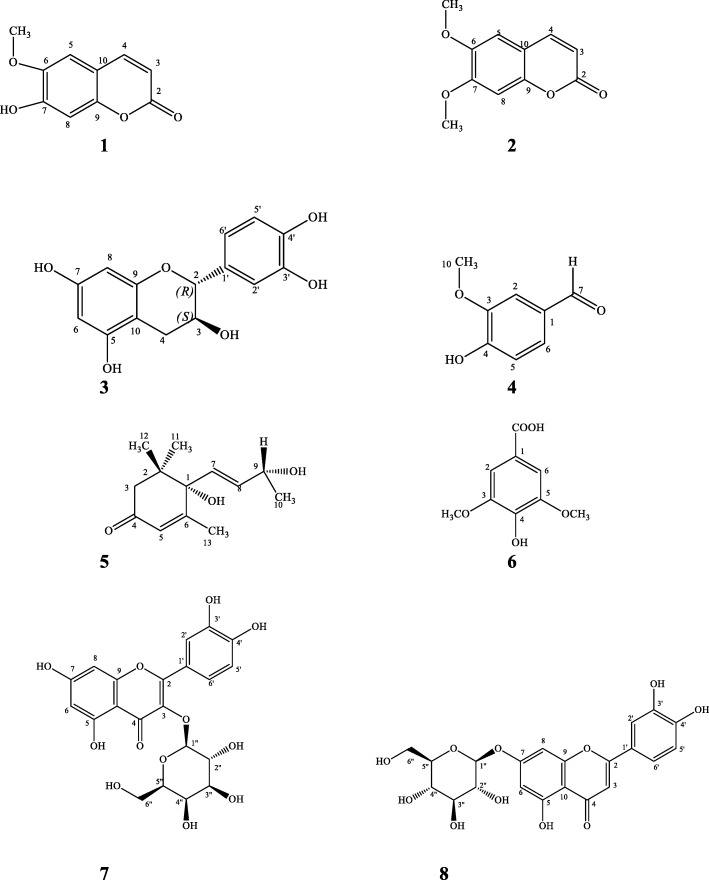
Bioactive compounds isolated from the water fractions of the ethanol extracts of the leaves of *Canarium patentinervium* Miq

Our team was the first to study and document the preliminary results of this rare plant. Our earlier studies on the crude extract of *C.patentinervium* Miq. have established its in vitro antioxidant, antitumor activity and antibacterial activity with the disc diffusion screening [7]. Three known compounds were isolated and identified from the chloroform fraction of the ethanol crude extract of leaves; compound 1 (scopoletin), compound 2 (scoparone), compound 3 (+)-catechin, while the chloroform extract of barks yielded three known compounds; compound 4 (lioxin), compound 5 (vomifoliol) and compound 6 (syringic acid) by comparing their NMR data with those in the literature (Fig. 1) [8, 9]. The current study is undertaken to further screen the comprehensive antibacterial activity of the extracts, fractions and isolated compounds from this plant. A bioassay guided fractionation was also undertaken in order to deeply evaluate the antibacterial activity of the water fraction of the ethanol extract of the leaves. We hope to provide further scientific evidence to the ethnopharmacology usage of this plant by investigating antibacterial properties of the plant and its isolated constituents.

## Methods

### Plant material

The leaves and barks of *C. patentinervium* Miq. were previously collected from one individual tree from Bukit Putih, Selangor, Malaysia (﻿3°5′24 “N 101°46′0”E). Plant was collected with the approval and assistance of the local indigenous people. The plant was identified by Mr. Kamaruddin (Forest Research Institute of Malaysia). A herbarium sample (PID 251210–12) has been deposited in the Forest Research Institute of Malaysia. The leaves and barks were air dried and grinded into small particles using an industrial grinder.

### Chemicals

Mueller Hilton broth (Becton Dickinson, Sparks, MD, USA), tryptic soy broth, and tryptic soy agar were purchased from (Bacto), petroleum ether and chloroform were purchased from Friendemann Schmidt Chemicals. Methanol and ethanol 95% were purchased from Kollin Chemicals. DMSO was purchased from R&M Marketing, Essex UK. INT (Iodonitrotetrazolium chloride), antibiotics amphotericin and ampicillin were purchased from Sigma Aldrich, Germany.

### Bacterial strains

The clinical isolates of bacteria were obtained from University Kebangsaan Malaysia Medical Centre (UKMMC). All bacterial strains were isolated from clinical specimens of hospitalized patients identified according to the Centers for Disease Control and Prevention/National Healthcare Safety Network (CDC/NHSN) criteria [10]. For all the experiments four different microbial (ATCC reference) cultures and ten clinical isolates were used (nine clinical isolates of bacterial and one clinical isolates of yeast):

Reference strains: *Staphylococcus aureus*, MSSA (ATCC 11632), *Bacillus cereus* (ATCC 10876), Methicillin-resistant *Staphylococcus aureus*, MRSA (ATCC 43300), *Pseudomonas aeruginosa* (ATCC 10145).

Clinical isolates: *Staphylococcus aureus* MSSA, *Staphylococcus aureus* MRSA, oxacillin-susceptible coagulase-negative *staphylococci*, oxacillin-resistant coagulase-negative *staphylococci*, *Enterococcus faecalis*, *Escherichia coli*, *Klebsiella* sp., *Escherichia coli* ESBL, *Klebsiella pneumoniae* ESBL. Yeast: *Candida parapsilosis.*

## Methods

### Bioassay-guided fractionation, isolation and identification of active compounds

#### Extraction, fractionation and isolation

Crude extraction was as previously done [7]. Dried and grinded sample of leaves and barks were soaked in hexane with the ratio of 1: 3 parts of sample to solvent for 2 h in a 60 °C water bath, filtered and concentrated with a rotary evaporator (Buchi, R-200 Switzerland). Thereafter, the leaves and barks were left to air dry completely for 3 days before repeating the whole process with chloroform, and then ethanol, respectively. The extracts were kept at − 20 °C till further use. Previously the ethanol extract of the leaves (80.0 g) was partitioned with petroleum ether, chloroform, and water to yield the respective solvent fractions. In the current study the water fraction (24.0 g) was further purified by bioassay guided fractionation using Sephadex LH-20 with mobile phase ethanol yielding 16 fractions which was then recombined to four fractions (WA, WB, WC, WD) according to the antibacterial activity. Subfraction WD was loaded on Sephadex LH-20 with mobile phase methanol yielding compound **7** (5.2 mg) and compound **8** (4.8 mg) (Fig. 1).

#### Main spectroscopic data for compounds 7–8

Hyperin (**7**) [11, 12]: Pale yellow powder: ^1^H-NMR (500 MHz, CD_3_OD), δ; 3.45 (H-5″, t, *J* = 9.0 Hz, 1H), 3.56 (H6b”, dd, *J* = 11.0, 3.0 Hz, 1H), 3.60 (H-3″, t, *J* = 9.0 Hz, 1H), 3.67 (H-6a”, dd, *J* = 11.0, 5.8 Hz, 1H), 3.84 (H-2″, t, *J* = 9.0 Hz 1H), 3.87 (H-4″, t, *J* = 9.0 Hz, 1H), 6.23 (H-6, d, J = 2.0 Hz, 1H), 6.43 (H-8, d, *J* = 2.0 Hz, 1H), 7.60 (H-6′, dd, J = 2.6, 8.6 Hz, 1H), 6.88 (H-5′, d, *J* = 8.5 Hz, 1H), 7.86 (H-2′, d, *J* = 2.0 Hz, 1H); ^13^C-NMR (125 MHz, CD3OD), δ; 60.53 (C-6″), 70.2 (C-4″), 74.0 (C-2″), 75.80 (C-3″), 77.88 (C-5″), 93.34 (C-8), 98.55 (C-6), 103.99 (C-1″), 104.17 (C-10), 114.67 (C-2′), 116.36 (C-5′), 121.51 (C-1′, C-6′), 134.36 (C-3), 144.43 (C-3′), 148.56 (C-4′), 157.08 (C-9), 157.37 (C-2), 161.63 (C-5), 164.90 (C-7).

Cynaroside (**8**) [13, 14]: Light yellow powder: ^1^H-NMR (500 MHz, CD_3_OD, δ), 7.60 (H-6′, dd, 1H, J = 2.6, 8.6 Hz,), 6.88 (H-5′, d, 1H, J = 8.6 Hz), 6.43 (H-8, d, 1H, J = 2.1 Hz), 6.23 (H-6, d, 1H, J = 2.1 Hz), 6.60 (H-3, s, 1H), 5.19 (H-1″, d, 1H, *J* = 8.2 Hz), 3.30 (H-4″, t, 1H, J = 9.0 Hz), 3.20 (H-2″, t, 1H, *J* = 9.0 Hz), 3.67 (H-6”a, dd, 1H, J = 11.0, 3.0 Hz), 3.56 (H-6”b, dd, 1H, J = 11.0, 5.8 Hz), 3.60 (H-3″, t, 1H, J = 9.0 Hz) and 3.45 (H-5″, t, J = 9.0 Hz, 1H); ^13^C-NMR (125 MHz, CD3OD),δ: 178.1 (C-4), 164.90 (C-7), 161.63 (C-5), 157.37 (C-2), 157.08 (C-9), 148.56 (C-4′), 144.43 (C-3′), 134.36 (C-3), 121.51 (C-1′, C-6′), 116.36 (C-5′), 114.67 (C-2′), 104.17 (C-10), 103.99 (C-1″), 98.55 (C-6), 93.34 (C-8), 77.88 (C-5″), 75.80 (C-3″), 73.77 (C-2″), 69.62 (C-4″), 60.53 (C-6″).

#### Evaluation of the antimicrobial activity

**Minimum inhibitory concentration (MIC***)* determination by microtitre broth dilution method: MIC was performed on all extracts, fractions and isolated compounds. The MIC of the plant extracts was determined by serial dilution, as described by Eloff [15] according to the Clinical and Laboratory Standards Institute (CLSI) guidelines. Stock solutions of the respective plant extracts were prepared in 1.5 ml microcentrifuge tubes (Eppendorff) by dissolving dry plant extract in dimethylsulphoxide (DMSO) to a final concentration of 64 mg/ml. The serial dilutions from the stock solution were made ranging from 32 mg/mL to 0.25 mg/mL using Mueller–Hinton broth (Becton Dickinson, Sparks, MD, USA) in 96-well microplates. The bacterial suspension containing approximately 5 × 10^5^ colony-forming units/mL was prepared from a 24 h culture plate. From this suspension, 100 μl was inoculated into each well. A sterility control well and a growth control well were also studied for each strain. The microtiter plates were incubated at 37 °C, 24 h for bacteria and 48 h for yeasts (as yeasts require a longer time for growth). After incubation at 37 °C, 40 μl of a 0.4 mg/ml solution of INT was added to each well as an indicator of microbial growth. The plates were incubated at 37 °C for 30 min (bacteria) and 24 h (yeast) and the MIC values visually determined. The lowest concentration of each extract displaying no visible growth was recorded as the minimum inhibitory concentration. The concentration that inhibited bacterial/yeast growth completely (the first clear well) was taken as the MIC value. MIC values were determined at least in duplicate and repeated to confirm activity.

In order to, determine the sensitivity of the microorganisms, positive control experiments were conducted: (1) for bacterial strains, ampicillin (Sigma-Aldrich) at a starting concentration of 0.10 mg/ml in sterile water, and (2) for yeast strains, Amphotericin B (Sigma-Aldrich), at a starting concentration of 0.10 mg/ml in DMSO and water (where 1.00 mg/ml was prepared in DMSO, and diluted to 0.10 mg/ml in sterile water thereafter). The final concentrations for these experiments ranged from 25.00 μg/ml (row A) to 0.19 μg/ml (row H). A negative control experiment was conducted using only DMSO. For isolated compounds, the serial dilutions from the stock solution were made ranging from to 500 μg/mL to 3.90 μg/mL using Mueller–Hinton broth (Becton Dickinson, Sparks, MD, USA) in 96-well microplates.

**Minimum bactericidal concentration (MBC)** determination by microtitre broth dilution method: Minimum bactericidal concentration (MBC) was recorded as a lowest extract concentration killing 99.9% of the bacterial inocula after 24 h incubation at 37 °C. The determination of MBC was performed using the method of Ozturk & Ercisli [16]. MBC was performed on all extracts, fractions and isolated compounds. Ten microliters were taken from the well obtained from the MIC experiment (MIC value) and two wells above the MIC value well and spread on MHA plates. The number of colony was counted after 18–24 h of incubation at 37 °C. The concentration of sample that produces < 10 colonies was considered as MBC value. Each experiment was repeated at least three times.

**Time-kill assay**: Compounds with the lowest MBC/MIC ratio are tested for the time kill behaviour [17] from Table 6. Compounds selected are scopoletin, scoparone, hyperin, cynaroside and syringic acid. Assay was performed using the microplate method by Datta et al. [18]. Due to insufficient yield of compounds isolated, all samples selected were tested for the antimicrobial effect only against MRSA clinical strain. Samples and inoculums were prepared and 100 μl of samples were added to the wells, followed by addition of 100 μl of microbial broth at concentration of 10 × 10^5^ colony forming units (cfu)/ml, grown in Mueller Hilton Broth (MHB) to each well. This yielded a final volume of 200 μl in each well of the extract. The plates were then incubated at 37 °C and optical density was recorded at 1 h intervals up to 18 h at wavelength of 600 nm. Graph was plotted for turbidity against time. The growth rate thus obtained was studied for any signs of bactericidal effects of the plant extract. Ampicillin was used as positive control. A solution of the solvent in which dried extract compound was dissolved served as negative control.

### Statistical analysis

Concentration–response curves were calculated using the Prism software package 5.00 for Windows, GraphPad Software, San Diego California USA, www.graphpad.com (GraphPad, San Diego, USA) and data were reported as mean and SD values obtained from a minimum of three determinations. Non-linear best fit was plotted with SD and 95% confidence interval. All data were expressed as mean ± standard deviation. Data were analyzed using one way Anova followed by Tukey test using GraphPad Prism5 software. A significant difference was considered at the level of *P* < 0.01. Time-kill data was plotted on Microsoft Excel.

## Results

### Minimum inhibitory concentration (MIC)

Results for the extracts and fractions are displayed in Table 1. The extract with best activity was chosen and fractionation was done. The leaves extract could be identified as the most active extract with MICs ranging from 0.25–2 mg/ml on 12 out of 14 microbial strains investigated [*Staphylococcus aureus* (ATCC 11632), *Bacillus cereus* (ATCC 10876), Methicillin-resistant *Staphylococcus aureus* (ATCC 43300), *Pseudomonas aeruginosa* (ATCC 10145), Clinical isolates; (*Staphylococcus aureus* MSSA, *Staphylococcus aureus* MRSA, Oxacillin-susceptible coagulase-negative *staphylococci*, oxacillin-resistant coagulase-negative *staphylococci*, *Enterococcus faecalis*, *Klebsiella* sp., *Klebsiella pneumoniae* ESBL. Yeast: *Candida parapsilosis*] (Table 1). The leaves extract also demonstrated highest antibacterial activity against clinical MRSA with MIC of 0.25 mg/ml. Strong microbial inhibitors possessed MIC values of equal or lower than 0.50 mg/ml [19, 20]; a clear indication that the MIC value of 0.25 mg/ml obtained for ethanol extracts (leaf and bark) against MRSA *Staphylococcus aureus* (clinical strain) indicates exceptional antimicrobial activity. The ethanol extract of leaves also resulted with the MIC of 0.50 mg/ml against MSSA (both strains) and MRSA (ATCC 43300). Oxacillin-susceptible coagulase-negative *staphylococi* and oxacillin-resistant coagulase-negative *staphylococci* were both sensitive against both ethanol extracts with an MIC of 0.5 mg/ml for the bark extracts and MIC range of 0.5 mg/ml to 1.00 mg/ml for the leaves.

**Table 1:** Minimum bactericidal concentration (MBC), minimum inhibitory concentration (MIC) and MBC/MIC ratio of *Canarium patentinervium* Miq. extracts (mg/ml) and positive control antimicrobial agents (μg/ml) against 4 ATCC bacteria and 9 bacteria and 1 yeast clinical isolates

Bacteria/ yeast	Plant extracts MBC values (mg/ml) MIC values (mg/ml) MBC/MIC ratio, (+) bactericidal; (−) bacteriostatic									Control antimicrobial agents MBC values (mg/ml) MIC values (mg/ml) MBC/MIC ratio	
	LH	LC	LE	BH	BC	BE	PF	CF	WF	Ampicilin	Amphotericin
MSSA ATCC 11632	–	–	2 0.5 4(+)	2 2 1(+)	1 1 1(+)	1 1 1(+)	4 0.5 8(−)	4 2 2(+)	1 0.5 2(+)	6.25 1.56 4(+)	NA
MRSA ATCC 43300	–	–	2 0.5 4(+)	4 1 4(+)	–	4 1 4(+)	2 0.5 4(+)	2 1 2(+)	1 0.5 2(+)	–	NA
*Bacillus cereus* ATCC 10876	–	–	1 0.5 2(+)	8 1 8(−)	–	1 0.5 2(+)	2 0.5 4(+)	2 1 2(+)	2 0.5 4(+)	> 25 6.25	NA
*Ps.aeruginosa* ATCC 10145	–	–	4 1 4(+)	8 4 2(+)	–	–	–	8 4 2(+)	2 1 2(+)	–	NA
MSSA	–	–	4 0.5 8(−)	4 2 2(+)	4 2 2(+)	2 0.5 4(+)	1 0.5 2(+)	4 2 2(+)	2 0.5 4(+)	> 25 > 25	NA
MRSA	–	–	2 0.25 8(−)	8 2 4(+)	–	1 0.25 4(+)	4 0.25 16(−)	4 2 2(+)	1 0.25 4(+)	–	NA
*Escherichia coli*	–	–	–	–	–	2 0.5 4(+)	–	–	–	–	NA
*Escherichia coli* ESBL	–	> 16/8	–	> 16/8	> 16/8	–	–	–	–	–	NA
Oxacillin-resistant CONS	–	> 16/8	4 1 4(+)	2 0.5 4(+)	8 2 4(+)	4 0.5 8(−)	–	2 0.5 4(+)	1 0.5 2(+)	–	NA
Oxacillin-sensitive CONS	> 16/16	> 16/8	2 0.5 4(+)	–	–	2 0.5 4(+)	–	–	1 0.5 2(+)	12.5 8 1.6(+)	NA
*Enterococcus faecalis*	–	–	4 0.5 8(−)	–	–	4 0.5 8(−)	–	–	2 0.5 4(+)	6.25 3.13 2(+)	NA
*Klebsiella* species	–	–	2 0.5 4(+)	16 4 4(+)	16 4 4(+)	2 0.5 4(+)	–	8 4 2(+)	1 0.5 2(+)	> 25 > 25	NA
*Kleb.pneumoniae* ESBL	–	–	2 0.5 4(+)	–	–	2 0.5 4(+)	–	–	1 0.5 2(+)	–	NA
*Candida parapsilosis*	–	–	8 2 4(+)	> 16 8	–	8 2 4(+)	8 2 4(+)	16 8 2(+)	4 2 2(+)	NA	3.13 0.78 4(+)

*LH* leaf hexane extract; *LC* leaf chloroform extract; *LE* leaf ethanol extract; *BH* bark hexane extract, *BC* bark chloroform extract, *BE* bark ethanol extract; *PF*petroleum ether fraction, *CF* chloroform fraction, *WF* water fraction, *CONS*, coagulase-negative staphylococci; *MRSA*, methicillin-resistant *Staphylococcus aureus; MSSA*, methicillin-sensitive *Staphylococcus aureus*

-, no activity; NA - not applicable

Upon fractionation of the ethanol extract of leaves, the water fraction was the most active fraction inhibiting 12 out of 14 bacteria tested with MICs ranging from 0.25 mg/ml to 2 mg/ml similar, to the ethanol extract. The more apolar petroleum ether and chloroform fractions seemed to be less active than the water fraction. The chloroform fraction also exhibited antibacterial activity on nine out of 14 bacteria tested with MICs ranging from 0.5–8 mg/ml. The petroleum ether fraction had positive activity on six out of 14 bacteria, with MIC ranging from 0.25–2 mg/ml. The chloroform and water fraction was most active against coagulase negative *Staph*: Oxacillin (R) with MIC of 0.5 mg/ml, showing superior activity than the whole extract. The petroleum ether and water fraction was active against MRSA at a concentration equal to the whole extract with MIC of 0.25 mg/ml.

### Minimum bactericidal concentration (MBC)

According to the ratio MBC/MIC, we appreciated antibacterial activity. If the ratio MBC/MIC ≤4, the effect was considered as bactericidal but if the ratio MBC/MIC > 4, the effect was defined as bacteriostatic [21, 22]. The MBC/MIC ratio are displayed in Table 1. The most promising activity was displayed against gram-positive bacteria *Staphylococcus aureus*, MRSA, *Bacillus cereus* and gram-negative bacteria *Pseudomonas aeruginosa, Klebsiella* sp., *Klebsiella pneumoniae* ESBL*.* The ethanol extracts of leaves and barks showed inhibition against *Candida parasilopsis* with a cidal activity. All the tested bacteria were susceptible to either bark or leaf extracts. The ethanol extracts (bark and leaves) were bactericidal against *Bacillus cereus* with a MIC of 0.50 mg/ml and MBC of 1.00 mg/ml. The ethanol extracts were also bactericidal against gram-negative *Klebsiella* sp. and *Klebsiella pneumoniae* ESBL with MIC of 0.50 mg/ml and MBC of 2.00 mg/ml.

All crude extracts of *C. patentinervium* Miq. under investigation exhibited exceptional concentration-dependent antimicrobial activity against both Gram-positive and Gram-negative bacteria. The most active fraction was the water fraction with consistently lower MBC than the extract. The most promising activity was displayed against gram-positive bacteria *Staphylococcus aureus*, MRSA, *Bacillus cereus* and gram-negative bacteria *Pseudomonas aeruginosa, Klebsiella* sp., *Klebsiella pneumoniae* ESBL.

### MIC, MBC and MBC/MIC ratio of isolated compounds

The antibacterial results for the previously isolated compounds (Compound 1–6) and currently isolated compound 7–8 are shown in Table 2. *Staphylococcus aureus* ATCC 11632 was sensitive against all compounds tested. Scopoletin had the best activity with MIC 25.00 ± 0.00 μg/ml and complete bacterial kill, MBC of 50.00 ± 0.00 μg/ml. Scoparone, (+) catechin, hyperin, cynaroside and syringic acid had bactericidal activity at 100.00 ± 0.00 μg/ml.

**Table 2:** MIC, MBC and MBC/MIC ratio for isolated compounds from *Canarium patentinervium* Miq. against *Staphylococcus aureus* ATCC 11632

Compounds	Concentration (μg/ml)		MBC/MIC ratio
	MIC	MBC	
scopoletin^a^	25.00 ± 0.00	50.00 ± 0.00	2 (+)
scoparone^a^	50.00 ± 0.00	100.00 ± 0.00	2 (+)
(+)-catechin^a^	50.00 ± 0.00	> 100	nd
Hyperin (water fraction)	50.00 ± 0.00	100.00 ± 0.00	2 (+)
Cynaroside (water fraction)	50.00 ± 0.00	100.00 ± 0.00	2 (+)
vomifoliol^a^	100.00 ± 0.00	> 100	nd
lioxin^a^	100.00 ± 0.00	> 100	nd
syringic acid^a^	50.00 ± 0.00	100.00 ± 0.00	2 (+)

*nd* not determined,

^a^previously isolated compounds from the chloroform fraction [8]

### Time-kill assay

**Fig. 2:**
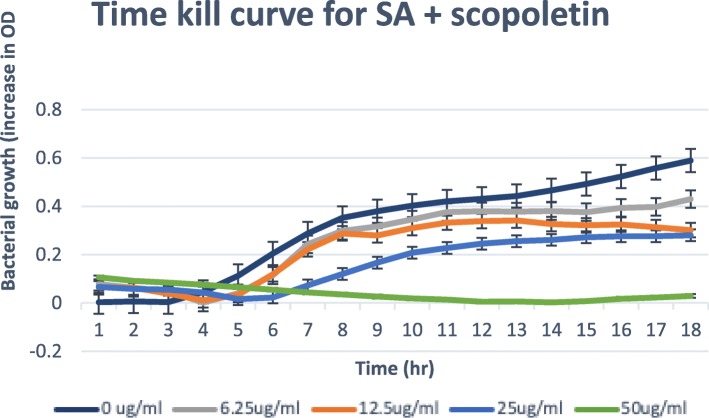
Time-kill plot for *Staphylococcus aureus* (SA) in the presence of scopoletin isolated from *Canarium patentinervium* Miq

**Fig. 3:**
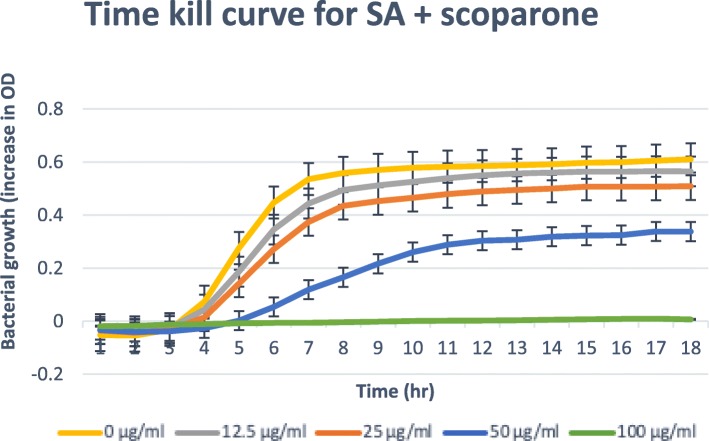
Time-kill plot for *Staphylococcus aureus* (SA) in presence of scoparone isolated from *Canarium patentinervium* Miq

**Fig. 4:**
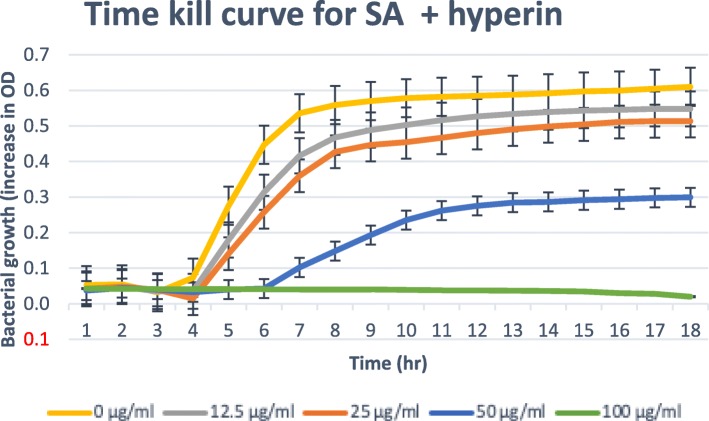
Time-kill plot for *Staphylococcus aureus* (SA) in presence of hyperin isolated from *Canarium patentinervium* Miq

**Fig. 5:**
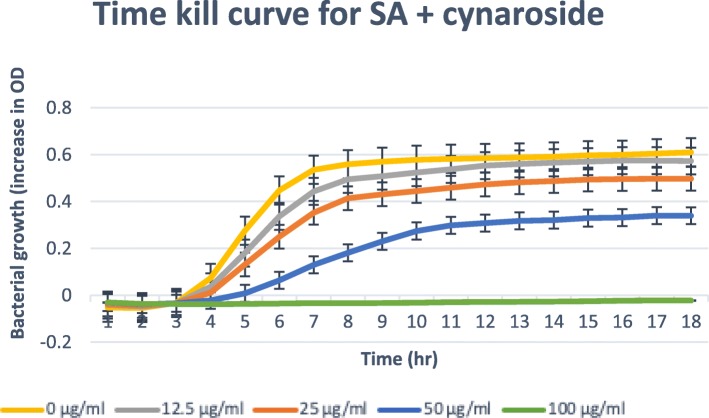
Time-kill plot for *Staphylococcus aureus* (SA) in presence of cynaroside isolated from *Canarium patentinervium* Miq

**Fig. 6:**
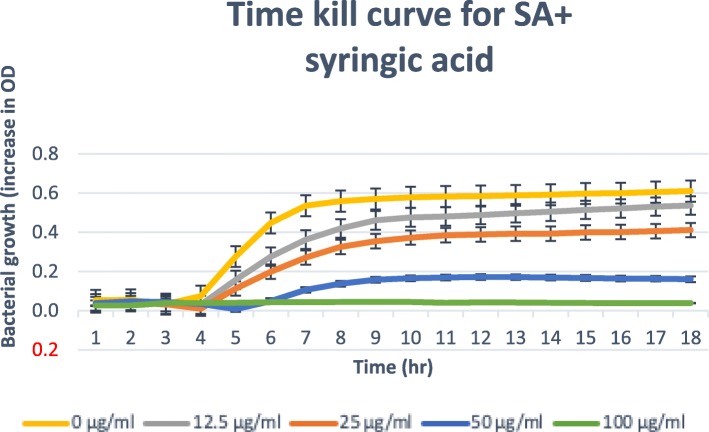
Time-kill plot for *Staphylococcus aureus* (SA) in presence of syringic acid isolated from *Canarium patentinervium* Miq

Only isolated compounds that have MBC/MIC ratio ≤ 4, (bactericidal) are tested for the time-kill [21, 22]. The results obtained for the time-kill study of *C. patentinervium* Miq. are shown in Fig. 2, 3, 4, 5 and 6. The MBC values was consistent with the cidal concentration as displayed in the growth curve of this time-kill study.

## Discussion

*C. patentinervium* Miq. is a rare plant from the family of Burseraceae and genus *Canarium* found in Asia Pacific region previously recorded for its usage in wound healing by the indigenous people of Malaysia [6]. Wound healing involves manifold inflammatory processes of which notably the massive release of leukotrienes from arachidonic acid via the 5-lipoxygenase pathway (5-LOX) and the generation of nitric oxide (NO) from inducible nitric oxide synthase (iNOS) [23, 24]. Of note, nitric oxide (NO) is a free radical, and the generation of cytokines involves a reactive oxygen species (ROS) outburst [25]. Therefore, agents able to block the enzymatic activity of 5-LOX and to scavenge free radical are of immense interest against inflammatory conditions and epidermal insults [26, 27]. Cells produce superoxide anion (O_2_^−)^, peroxide anion (HO_2_^−)^, and hydroxyl ion (HO^−^) as part of the physiological aerobic metabolism which are quickly scavenged by cytoplasmic antioxidant defense system [25]. Once skin is injured, micro-organisms that are normally sequestered at the skin surface obtain access to the underlying tissues of which *Staphylococcus aureus* appear to play an important role in bacterial infection in wounds [28]. The anti-inflammatory and antioxidant activities were previously evaluated with promising results [7, 29].

Our preliminary antibacterial studies with disc diffusion method suggested an interesting profile of antimicrobial action against gram positive and gram negative bacteria [7]. In this study, a comprehensive investigation was done to evaluate the total bactericidal/ bacteriostatic activity of an extract and its fraction against a range of reference and clinical isolates of bacteria obtained from a local hospital using the MIC, MBC, MBC/MIC ratio and time-kill assay. The majority of the crude bark and leaf extracts of *C.patentinervium* Miq. exhibited moderate to relatively good activity against gram-positive and gram-negative pathogens with the MIC values ranging from 0.25 mg/ml to 16.00 mg/ml depending on the individual extract, fraction and bacteria tested (Table 1). Only the hexane extract of leaves exhibited weak antimicrobial activity. All active extracts displayed concentration-dependent antimicrobial activity. Aligiannis et al. [30] proposed a classification system based on MIC results obtained for plant materials, which was consequently described and implemented by Duarte et al. [20]. All plant species with MIC values of up to 8 mg/ml are considered to possess at least some degree of inhibitory effect, and any concentration exceeding this should not be considered effective, according to Fabry et al. [31]. Moderate microbial inhibitors are described by Aligiannis et al. [30] as those plant extracts with MIC values ranging between 0.60 mg/ml and 1.50 mg/ml.

Amongst the extracts investigated in the present study, hexane extracts of the barks seemed to have most of the moderate inhibitors with an interesting bactericidal action against MRSA ATCC 43300 strain (MIC of 1.00 mg/ml and MBC of 4.00 mg/ml). Weak microbial inhibitors are classified as those agents with MIC values of between 1.60 mg/ml and 8.00 mg/ml [30]. None of the extracts in the present study yielded MIC values in excess of 8.00 mg/ml against gram-positive bacteria except with hexane extract of leaves (Additional file 1).

In general, the gram-negative bacteria displayed the least sensitivity towards the extracts and fractions, and most of the plant extracts (except ethanol extract of barks) exhibited poor and unvaried activity against *Escherichia coli*, indicating the resistance of this bacterium to the plant extracts. This was to be expected, as gram-negative bacteria offer a much more complex barrier system against permeation of foreign substances (in this case, the antimicrobial agent). This is attributed to the specialized cell wall structure and especially the presence of the outer envelope resulting in the impermeability of these micro-organisms to biocides and antibiotics, and at times, resulting in regulation and prevention of their passage to the target region [32].

Resistance to the plant extracts is, thus, exhibited to a far greater extent by the gram- negative bacteria than by gram-positive bacteria [33]. The lipophilic or hydrophilic nature of compounds also plays a role in the activity, or lack thereof, against the microorganisms. Compounds considered to be more effective against gram-negative bacteria are considerably less lipophilic. This is because of the structure of the gram-negative cell wall which also has a higher lipid content [34]. The outer layer of the gram-negative outer membrane is composed of lipopolysaccharide molecules that provides hydrophilic environment that gives protection against hydrophobic molecules [35]. This could explain why in this study the ethanol extracts and its water fraction which contain more hydrophilic compounds showed more inhibition against gram-negative bacteria such as *Klebsiella* species.

The ethanol extracts also have shown to contain polyphenols such as tannins and flavonoids from our previous phytochemical analysis [7]. Polyphenols, such as tannins and flavonoids, have important antibacterial activity [36]. The antimicrobial activity of flavonoids is due to their ability to complex with extracellular and soluble protein and to complex with bacterial cell wall while that of tannins may be related to their ability to inactivate microbial adhesions, enzymes and cell envelop proteins [37]. Flavones are phenolic structures containing one carbonyl group as opposed to the two carbonyls in quinones. The addition of a 3-hydroxyl group yields a flavonol. Flavonoids are also hydroxylated phenolic substances but occur as a C6-C3 unit linked to an aromatic ring. Their activity is probably due to their ability to complex with extracellular and soluble proteins and to complex with bacterial cell walls [38].

Many human physiological activities, such as stimulation of phagocytic cell, host mediated tumour activity and a wide range of anti- infective actions, have been assigned to tannins. Their mode of antimicrobial action may be related to their ability to inactivate microbial adhesions, enzymes, cell envelope, transport-proteins etc. They also form a complex with polysaccharide. According to some studies, tannins can also be toxic to filamentous fungi, yeasts and bacteria [36]. However, it is important to note that, if tannins were solely responsible for the activity presented by these results, this activity would be observed against all organisms and would not be limited to gram-positive bacteria or yeasts. The current hypothesis is thus that tannins are at least partially responsible for the antibiotic activity [37].

All isolated compounds tested against *Staphylococcus aureus* ATCC 11632 showed bacterial growth inhibition. Scopoletin, scoparone, hyperin, cynaroside and syringic acid had bactericidal effect at ≤100 μg/ml. The graphical abstract for the isolated compounds and activities are shown in the supplementary file. Only scopoletin had bactericidal effect and complete kill at MBC 50.00 μg/ml (Fig. 2). It has then been suggested that coumarins possess antibacterial activity act selectively against gram positive microorganisms [39]. Free hydroxyl group at position 7 has been shown to be important for antibacterial activity [40] as confirming the activity of scopoletin that has free 7-OH. The antibacterial activity of compounds against other bacteria were not assessed due to the low quantity of the isolated compound. Two compounds isolated in this study are hyperin (**7**) which is a flavonol with a glucosidic moiety and cynaroside (**8**) which is a flavone with a glycosidic moiety.

Hyperin has been reported to have antioxidant activity [41], inhibition of lipid peroxidation in rat liver microsomes [42] and protective effects to PC12 cells against cytotoxicity induced by hydrogen peroxide and tert-butyl hydroperoxide [43]. Cynaroside has been reported for moderate inhibition of enzymes for the synthesis of thromboxane B2 and leukotriene B4 as well as hydrogen peroxide scavenging activity, scavenge reactive oxygen and nitrogen species [44] to chelate transition metals, antibacterial [45], antiviral [46] and antifungal [47] activity. Thus, the current antibacterial activity seen here can be related to the previous reported activity of the compounds that directly relates to processes beneficial in wound healing.

These results provided a rather comprehensive profile of the bioactivities of the main constituents of LE. Among these compounds, scopoletin, scoparone, hyperin, cynaroside and syringic acid were found to have significant antibacterial activities. Scopoletin, a coumarin (known as 1,2-benzopyrones) consists of fused benzene and pyrone ring, is an important group of low molecular weight phenolics and have been widely used for prevention and treatment of various diseases. Hydroxycoumarins have attracted intense interests in recent years because of their diverse pharmacological properties. The results obtained against the gram-positive and gram-negative bacteria thus support the traditional use of *C.patentinervium* Miq. in wound healing and hold potential in the treatment of colds, wound healing and as an antiseptic.

## Conclusion

The bioassay-guided fractionation performed on the water fraction of the ethanol extract of leaves of *C.patentinervium* Miq. led to the isolation of hyperin and cynaroside as the antibacterial compound of this medicinal plant. These bioactive compounds and others isolated from our previous study may support the traditional use of *C. patentinervium* Miq. leaves for the treatment of wounds and hold potential in the treatment of infection and as an antiseptic. As modern cultures and scientific advances spread around the world, the depth knowledge forfeited may not be realised. It is thus important that the knowledge be documented and the traditional use be given some credence through modern scientific studies. *C. patentinervium* Miq. is such an example.

## Supplementary information

**Additional file 1.** MIC, MBC and MBC/MIC ratio for isolated compounds from *Canarium patentinervium* Miq against *Staphylococcus aureus* ATCC 11632

## Data Availability

The datasets used and/or analysed during the current study available from the corresponding author on reasonable request.

## Abbreviations

ATCC: American Type Culture Collection
PC12: Cell line derived from a pheochromocytoma of the rat adrenal medulla
