# Supplementary material for: Antibacterial activities of the extracts, fractions and isolated compounds from Canarium patentinervium Miq. against bacterial clinical isolates
Source: BMC Complement Med Ther. 2020 Feb 14;20:55. doi: 10.1186/s12906-020-2837-5 (PMC7076860; doi:10.1186/s12906-020-2837-5)
Supplement: Supplementary file 1 — Additional file 1. MIC, MBC and MBC/MIC ratio for isolated compounds from Canarium patentinervium Miq against Staphylococcus aureus ATCC 11632 [file 12906_2020_2837_MOESM1_ESM.pdf]

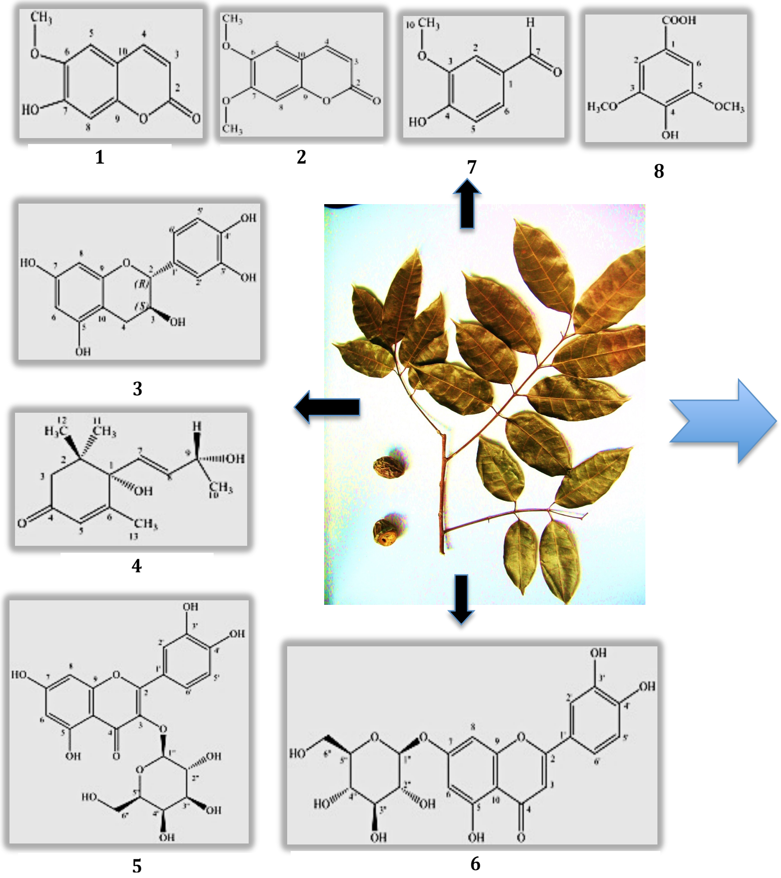

MIC, MBC and MBC/MIC ratio for isolated compounds from *Canarium patentinervium* Miq. against *Staphylococcus aureus* ATCC 11632

Compounds	Concentration (µg/ml)		MBC/MIC ratio
	MIC	MBC	
Compound 1- scopoletin[a]	25.00±0.00	50.00±0.00	2 (+)
Compound 2-scoparone[a]	50.00±0.00	100.00±0.00	2 (+)
Compound 3- (+)-catechin[a]	50.00±0.00	>100	nd
Compound 4- lioxin[a]	100.00±0.00	>100	nd
Compound 5- vomifoliol[a]	100.00±0.00	>100	nd
Compound 6- syringic acid[a]	50.00±0.00	100.00±0.00	2 (+)
Compound 7-Hyperin (water fraction)	50.00±0.00	100.00±0.00	2 (+)
Compound 8-Cynaroside (water fraction)	50.00±0.00	100.00±0.00	2 (+)

nd: not determined,
a: previously isolated compounds from the chloroform fraction (Mogana et al., 2014)
